# Bimanual movements in children with cerebral palsy: a systematic review of instrumented assessments

**DOI:** 10.1186/s12984-023-01150-7

**Published:** 2023-02-27

**Authors:** Marine Cacioppo, Anthéa Loos, Mathieu Lempereur, Sylvain Brochard

**Affiliations:** 1 Department of Physical Medicine and Rehabilitation, Brest University Hospital, 2 Avenue Foch, 29200 Brest, France; 2 Laboratoire de Traitement de L’information Médicale (LaTIM), Inserm U1101, Université de Bretagne-Occidentale, 29200 Brest, France; 3 Pediatric Rehabilitation Department, Fondation ILDYS, 29200 Brest, France; 4 Pediatric Rehabilitation Department, University Hospital of Rehabilitation (HU2R), Strasbourg, France

**Keywords:** Children, Cerebral palsy, Bimanual, Upper limb, Motion analysis, Instrumented measures, Assessments

## Abstract

**Background:**

Assessment of bimanual movements, which are frequently impaired in children with cerebral palsy, is highly challenging in clinical practice. Instrumented measures have been developed to evaluate and help to understand impaired upper limb movement during bimanual tasks in these children. The aim of this review was to report instrumented measurement tools (3D motion analysis, sensors, etc.) used for bimanual task movement analysis, and the metrological properties of the measures in children with cerebral palsy.

**Methods:**

A systematic review was conducted (Prospero CRD42022308517). PubMed, Web of Science, Cochrane and Scopus databases were searched with relevant keywords and inclusion/exclusion criteria. Article quality and biomechanical methods were evaluated with a customized scale and metrological properties with the COSMIN checklist.

**Results:**

In total, 452 children, mostly with unilateral cerebral palsy, mean age 10.9 (SD 3.2) years, underwent quantitative bimanual assessments in the 31 included studies (mean quality score 22/32 points [SD 4.7]). The tools used were 3D motion analysis (n = 26), accelerometers (n = 2), and other instruments (cube, digitizer, etc.) (n = 3). Children performed 1–5 bimanual tasks in laboratory settings, mostly activities of daily living or game scenarios. Analyses focused mostly on spatiotemporal variables, 6 of which were specifically developed for bilateral measures (task completion time, goal synchronization, movement overlap time, interlimb coupling, continuous relative phase and asynchrony). These instrumented measurements had moderate to good discriminant and convergent validity, but reliability and responsiveness assessments were lacking.

**Conclusions:**

A large number of quantitative bimanual assessments involving different tools, bimanual tasks and specific variables developed to evaluate bimanual function were found. Development of other relevant variables and validation of these tools are needed to further determine their usefulness, both as research outcomes and to guide therapies in clinical practice. Future research, involving younger children and real-life assessments, will improve our understanding of bimanual function in children with cerebral palsy.

**Supplementary Information:**

The online version contains supplementary material available at 10.1186/s12984-023-01150-7.

## Background

Most daily activities require both hands (e.g., buttoning a shirt, tying laces and opening a bottle). For children with cerebral palsy (CP), accomplishing these daily activities can be challenging because of the motor deficit, limited range of motion, lack of motor control and spasticity of their impaired upper limb (UL) [1–3]. Bimanual movements are defined as both hands working together to achieve a goal. They are more complex than unimanual movements since they involve coordination of both ULs, with coupling of the movement amplitude and direction of both hands (spatial and temporal constraints) [4, 5]. Difficulty executing bimanual tasks is one of the greatest causes of functional impairment for these children because it impacts their participation and quality of life [6–8]. Furthermore, since new, innovative therapies focus on developing and training bimanual performance to improve participation in daily life activities [9], validated evaluation of bimanual movements has become crucial.

Instrumented measurements of UL movements are being developed [10]. 3D movement analysis (3DMA), which is now used routinely for gait analysis [11], is becoming increasingly used for UL analysis, and typical measures include spatiotemporal and kinematic variables. However, 3DMA is mostly used to assess unimanual movements that do not reflect real life since most everyday tasks require the cooperative use of both hands [12]. Few protocols have been developed to assess spontaneous use of the impaired UL in bimanual conditions. The main challenge relating to such protocols performed in a laboratory setting is choosing appropriate bimanual movements that capture how children really move in their daily environment and that are representative of the large number of possible UL movements. Other, child-friendly tools are being increasingly developed to evaluate spontaneous and representative UL use. For example, accelerometers have many advantages (low cost, small size, accurate measurement, etc.), in particular, they can be used out with the laboratory setting in the child’s own environment [13].

Bimanual movement assessment with these quantitative tools is promising, increasingly used and required by both the research and clinical communities. However, a review of available tools, protocols, variables and metrological validation is lacking. Such a review would improve the understanding of impaired movements and would help to more accurately tailor interventions in children with CP. Moreover, few measures have been validated, although this is necessary to determine what is really measured.

The purpose of this systematic review was therefore to report instrumented tools (3DMA, sensors, etc.) and the metrological properties of the measures used to evaluate bimanual movements in children with CP. This systematic review aimed to (1) describe the types of instrumented tools used; (2) identify how data were collected and analyzed (protocols and variables); and (3) report the available evidence of validation of these measures (convergent and discriminant validity, within/between reliability and responsiveness) and (4) identify relevant outcomes for research and clinical application. Based on this review, we wished to draft a set of recommendations for the instrumented measurement of bimanual movements for future clinical development and research.

## Methods

### Reglementary issues

This systematic review is reported according to the PRISMA guidelines. A PRISMA checklist was completed and the review protocol was published in Prospero (CRD42022308517).

### Identification and selection of studies

Searches were conducted in English in the following databases: PubMed (1996 to March 2022), Scopus (2004 to March 2022), Cochrane Library (1995 to March 2022) and Web of Science (2004 to March 2022). To ensure the search was exhaustive, the following combinations of keywords were used: (1) keywords relative to children and adolescents: “child”, “adolescent”, “teen”, “infant”, “baby”, “newborn”; (2) “upper limb”, “upper extremities”, “upper body”, “arm”, “hand”, “bimanual”, “interlimb”; (3) “movement”,”motion”, “motor”, “biomechanical”, “kinematic”, “instrumented measurement”, “inertial”, “sensors”, “accelerometer”, “technologies”, “spatiotemporal”, “temporo-spatial”, “smoothness”, “fluidity”, “trajectory”; 4) “Cerebral Palsy”, “hemiplegia”, “hemiparesis”, “quadriplegia”, “tetraplegia”, “stroke”, “cerebrovascular accident” (the detailed equation is provided in Additional file 1). Search strings were formulated and tailored to the search syntax of each database to ensure a common search strategy.

Inclusion criteria were: (1) Design: full papers; (2) Participants: children and adolescents 0–18 years old with uni- or bilateral cerebral palsy; (3) Construct: exploration of bimanual movements (i.e. both hands used to achieve a goal) during symmetrical or asymmetrical tasks; (4) Type of instruments: quantitative instruments (3DMA, accelerometers, etc.); (5) Outcome measures: quantitative/instrumental measures of UL characteristics (spatiotemporal and/or kinematics and/or quality of movement variables and/or accelerometry) (see Additional file 2). Exclusion criteria were: (1) studies that evaluated spontaneous UL use (either unimanual or bimanual; no specific task) with no information on bimanual movements in the results section; (2) studies out of scope or not relating to motion analysis (e.g., electromyography or only force assessment); (3) conference papers; and (4) studies not published in English. Studies that included participants older than 18 years were also included, even if it was not possible to dissociate the results of the adults from those of the children and adolescents. Studies that included both typically developing children (TDC) and children with CP (unilateral or bilateral) were included, but the data from the TDC were not analyzed.

Two independent reviewers screened the titles and abstracts of the selected papers for inclusion. In case of disagreement, the full article was read and discussed until a consensus was reached.

### Assessment of characteristics of studies

The two reviewers extracted all the data independently.

#### Quality assessment

##### Intrinsic quality of articles and biomechanical methods (Q-score)

Study quality was assessed using a customized quality assessment scale developed from other scales in the literature used in the field of biomechanics or radiology [14–16]. The aim of the scale was to assess the intrinsic quality of each article and the quality of the biomechanical method using a Q score from 0 to 32 (Additional file 3). The first part of the scale was based on previously published quality checklists for systematic reviews as well as scales for the assessment of the quality of studies included in systematic reviews [17–19]. It included questions relating to study design and the quality of the reporting of methodologies and results, for example, “were the aims clearly stated?”. The second part of the scale related to the quality of the biomechanical protocol description, data acquisition and analysis, based on a previous scale used in this field [20] and other fields [14–16]. The quality rating was carried out independently by two reviewers (MC and AL) and disagreements were resolved by consensus.

##### Quality of metrological properties (COSMIN score)

For studies that specifically evaluated metrological properties, the Consensus based Standards for the selection of health Measurement Instruments (COSMIN) score risk of bias checklist 2017 [21, 22] was used to calculate a quality score for each metrological property. In this checklist, 3 boxes (“Hypotheses testing for construct validity”, “Reliability” and “Responsiveness”) were used. Each item in the boxes was rated as “very good” (3 points), “adequate” (2 points), “doubtful” (1 point) or “inadequate” (0 points). Convergent validity was assessed with Box 9.a (4 items, score/12), discriminant validity with Box 9.b (3 items, score/9), reliability with Box 6 (3 items, score/9) and responsiveness with Box 10.a (3 items, score/9).

#### Participants

Number of children and adolescents and demographics (age, sex, type of CP, topography of motor disorders, Manual Ability Classification System [MACS], etc.) were extracted.

#### Type of instruments

The following motion analysis data were extracted: type of instruments used, model; number, size and position of markers or sensors; sampling frequency (Hz); biomechanical model; specific algorithms and data analysis; protocols performed; starting position of the child; number of trials/sessions; velocity; duration of the protocol. The metrological properties (reliability, validity, responsiveness) were collected for each study. Construct validity was evaluated, including convergent validity (correlation with a gold standard measure or comparison with other outcome measurement instruments), and discriminant validity (ability to discriminate children with CP from another population). Articles in which children with CP were compared to TDC were considered to assess discriminant validity. Within/between rater/session reliability (ability of a test to provide the same measurement twice, e.g., intra-trial, inter-trial, test–retest) was evaluated with the measurement error and responsiveness (ability to detect a change before and after therapy). Studies that determined pre- and post-therapy effects and did not specifically assess the responsiveness of the measure were not considered to assess responsiveness.

#### Outcome measures

The following outcome measures were explored: spatiotemporal (velocity, duration, acceleration, distance, etc.), kinematic (angular values for the trunk, shoulder, elbow and wrist), quality of movement (smoothness, straightness, etc.) and actimetry (intensity and time). Variables measuring unilateral movement (only the affected UL during bimanual movements) and bilateral measures (both UL during bimanual movements) were distinguished.

### Data analysis

A descriptive analysis of samples, type of instruments and outcome measures was performed. Quantitative results are expressed as mean (standard deviation [SD]) and categorical results as number (%). We did not conduct a meta-analysis because of the large number of protocols, heterogeneity of samples and varied outcome measures that prevented such an analysis.

## Results

### Flow of studies

**Fig. 1:**
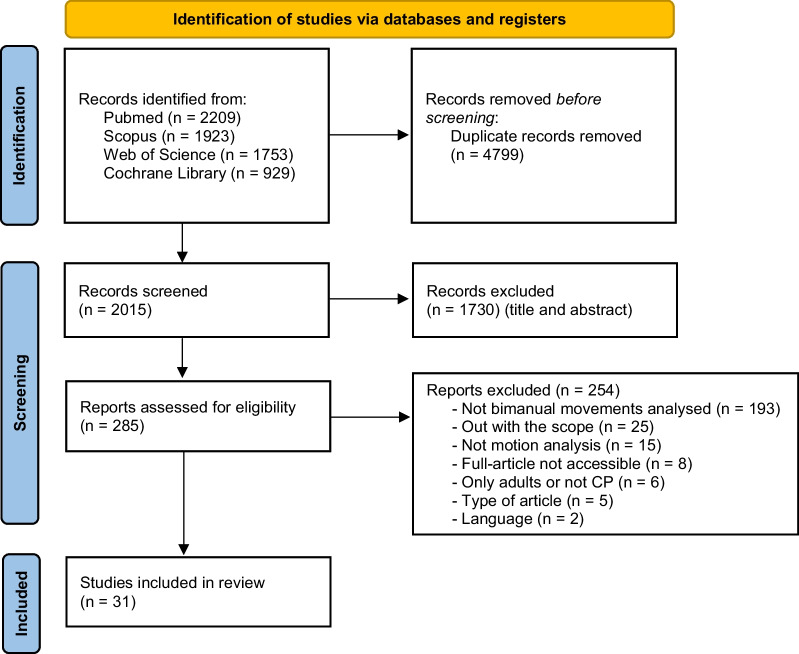
Flow diagram

The initial search identified 2015 papers published since 1995 after removal of duplicates. Following screening of titles and abstracts, 285 articles were deemed appropriate for full text screening. Of the 285 articles reviewed, 254 were excluded. The remaining 31 were included in the review (Fig. 1).

### Characteristics of included studies

**Fig. 2:**
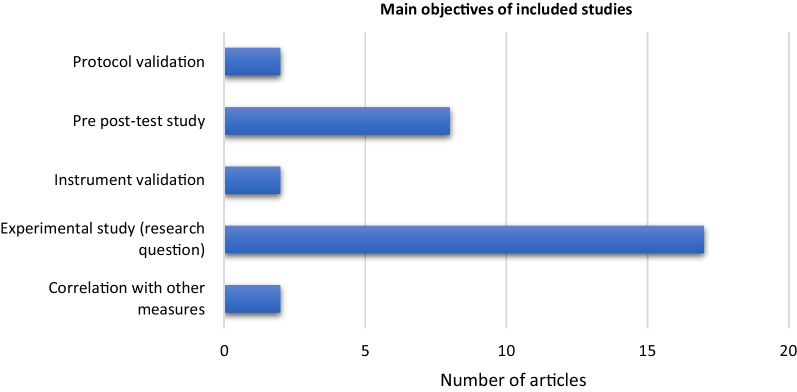
Main objectives of the 31 included studies

The aim of the studies was mostly to increase understanding of bimanual movement in different conditions (Fig. 2).

### Quality assessment

The mean Q-score of the articles included was 22/32 (SD 4.7). Seven articles had a score above 80% [23–29], nineteen articles had a score between 60 and 80% [30–47] and six articles had a score between 60 and 40% [48–53]. Details of the Q-scores of each article are provided in Additional file 4.

### Participants

In total, 452 children and young adults with CP, aged from 2 to 25 years (mean age 10.9 years [SD 3.2]) performed bimanual movements in the 31 studies and were included (Table 1). The majority had unilateral CP (uCP), only 3 children had bilateral CP [32, 41]. They were mainly female (318 females/241 males), with right-side impairment (161 left/181 right) and MACS level from I to III. Children under 5 years of age were included in 5/32 studies [23, 26, 35, 38, 49]). Children with uCP and TDC of same ages were included in 14 studies [23–25, 31, 33, 34, 36, 37, 39, 41–43, 47, 48]. One study compared children with uCP to an adult group [39].

**Table 1:** Overall description of included studies

Study	Design	Participants	Type of instrument	Outcome measures	Intervention
Beani et al. 2020 [23]	Case control study	N = 50 (+ 50 TDC) Mean age (range) = 9.9 (3–25) Classification = uCP, MACS I/II/III	Accelerometers	• Clinical scores: MACS • Quantitative measures: Asymmetry index, Mean activity count	
Cacioppo et al. 2020 [24]	Case control study	N = 19 (+ 20 TDC) Mean age (range) = 11.3 (5–17) Classification = uCP, MACS I/II/III	3DMA	• Clinical scores: AHA • Quantitative measures: ROM MAX APS SPARC IOC	
Cope et al. 2010 [30]	Experimental study	N = 10 Mean age (range) = 11 (7–14) Classification = uCP	3DMA	• Clinical scores: – • Quantitative measures: Peak velocity, Movement time, Movement units, ROM	CIMT
Feltham et al. 2010a [31]	Case control study	N = 8* (+ 14 TDC) Mean age (range) = 13.9 (9–18) Classification = uCP	3DMA	• Clinical scores: – • Quantitative measures: Duration, Interlimb coupling, Continuous relative phase, Normalized jerk	
Gaillard et al. 2019 [25]	Case control study	N = 20 (+ 20 TDC) Mean age (range) = 12 (6–18) Classification = uCP, MACS I/II/III	3DMA	• Clinical scores: AHA, Abilhand Kids • Quantitative measures: Angular waveforms, ROM MAX	
Gordon et al. 2007 [26]	Experimental study	N = 20 Mean age (range) = 7.7 (3–15) Classification = uCP	Accelerometers & 3DMA	• Clinical scores: – • Quantitative measures: Time of use, Goal synchrony	HABIT/ Usual therapy
Howcroft et al. 2012b [32]	Cohort study	N = 17 Mean age (SD) = 9.4 (1.5) Classification = uCP (15), bilateral CP (2)	3DMA	• Clinical scores: – • Quantitative measures: ROM, Angular velocities, Accelerations	
Hung et al. 2004 [33]	Cohort study	N = 10** Mean age (range) = 13.4 (8–16) Classification = uCP	3DMA	• Clinical scores: JTHFT • Quantitative measures: Task completion time, Goal synchronization, Movement overlap time, Tangential velocity, Peak velocity difference	
Hung et al. 2010 [34]	Case control study	N = 11** (+ 11 TDC) Mean age (range) = 13.1 (8–16) Classification = uCP	3DMA	• Clinical scores: – • Quantitative measures: Tangential velocity, Task completion time, Goal synchronization, Movement overlap, Task hand movement time	
Hung et al. 2011 [35]	Experimental study	N = 20 Mean age (range) = 6.9 (4–10) Classification = uCP, MACS I/II	3DMA	• Clinical scores: AHA • Quantitative measures: Peak tangential velocity, Movement overlap time, Goal synchronization, Task completion time	HABIT/CIMT
Hung et Meredith, 2014 [36]	Case control study	N = 10 (+ 10 TDC) Mean age (range) = 8.3 (7–11) Classification = uCP, MACS I/II	3DMA	• Clinical scores: – • Quantitative measures: Difference in vertical position between hands, Vertical hand ROM, Lateral hand ROM, Elbow excursion, Shoulder excursion	
Hung et al. 2017a. [27]	Experimental study	N = 20 Mean age (SD) = 8.5 (1.5) Classification = uCP, MACS I/II/III	3DMA	• Clinical scores: – • Quantitative measures: Movement overlap time, Goal synchronization, Task completion time, C7 displacement, Upper arm and elbow joint angle excursion	Structured/ unstructured practice
Hung et Spingarn, 2018 [37]	Experimental study	N = 7 Mean age (range) = 3.6 (2.4–4.5) Classification = uCP, MACS I/II/III	3DMA	• Clinical scores: – • Quantitative measures: Movement overlap time, Goal synchronization, Task completion time, C7 displacement, Upper arm and elbow joint angle excursion	H-HABIT
Hung et al. 2018 [38]	Case control study	N = 12 (+ 12 TDC) Mean age (range) = 8.3 (6–11) Classification = uCP, MACS I/II	3DMA	• Clinical scores: / • Quantitative measures: Movement time, Two hands offset/onset difference, Elbow/shoulder joint excursion	
Hung et al. 2019 [28]	Cohort study	N = 39 Mean age (range) = 9.6 (6–17) Classification = uCP, MACS I/II/III	3DMA	• Clinical scores: AHA • Quantitative measures: Total movement time, Goal synchronization, Normalized movement overlap, C7 displacement	
Hung et Zeng., 2020 [39]	Case control study	N = 10 (+ 10 TDC + 10 adults) Mean age (range) = 9.6 (6–11) Classification = uCP, MACS I/II	3DMA	• Clinical scores: – • Quantitative measures: Peak velocity, Upper arm and elbow joint, C7 displacement, Hand height position differences, Timing differences at offset	
Johansson et al. 2012 [40]	Case report	N = 2 Age = 17y and 13y Classification = uCP, MACS I/II	3DMA	• Clinical scores: – • Quantitative measures: Duration of the movement, 3D Distance, Number of movement units	SMT
Johansson et al. 2014 [41]	Case report	N = 1 (+ 1 TDC) Age = 12y Classification = uCP, MACS II	3DMA	• Clinical scores: – • Quantitative measures: 3D Distance, Number of movement units	SMT
Klotz et al. 2014 [42]	Case control study	N = 16 (+ 17 TDC) Mean age (range) = 13 (9–17) Classification = uCP, MACS I/II/III	3DMA	• Clinical scores: MACS, Abilhand Kids • Quantitative measures: Movement time, ROM	
Mutalib et al. 2019a. [43]	Case control study	N = 15 (+ 17 TD) Mean age (range) = 8.7 (5–14) Classification = uCP	A bespoke instrumented cube	• Clinical scores: – • Quantitative measures: Duration, Isometric grasp force, Interlimb force asymmetry, SPARC	
Rudisch, et al. 2016 [29]	Cohort study	N = 37 Mean age (SD) = 10.9 (2.6) Classification = uCP, MACS I/II/III	3DMA	• Clinical scores: CHEQ, JTHFT • Quantitative measures: Total task duration, Temporal Coupling, Spatial accuracy	
Shum et al. 2020 [49]	Experimental study	N = 5 (+ 12 TDC) Mean age (range) = 17 (14–21) Classification = uCP, MACS I/II/III	Kinect	• Clinical scores: *MACS • Quantitative measures: ROM, Peak velocity, Time to peak velocity, Smoothness, Trunk compensation	With / without EA
Smorenburg et al. 2011 [44]	Cohort study	N = 10* Mean age (range) = 12.7 (7–17) Classification = uCP	3DMA	• Clinical scores: – • Quantitative measures: Continuous relative phase	
Smorenburg et al. 2012a [45]	Cohort study	N = 23 Mean age (range) = 14.2 (9–19) Classification = uCP, MACS I/II/III	3DMA	• Clinical scores: – • Quantitative measures: Absolute error, Average velocity, Relative movement smoothness	
Smorenburg et al. 2013 [46]	Experimental study	N = 16 Mean age (range) = 15.8 (10–19) Classification = uCP, MACS I/II/III	3DMA	• Clinical scores: – • Quantitative measures: Absolute error	Visual / mirror feedback
Sugden et Utley, 1995 [49]	Cohort study	N = 17 Mean age (range) = 9.8 (4–18) Classification = uCP	3DMA	• Clinical scores: – • Quantitative measures: Duration, Mean/peak velocity, Location of peak velocity, Interlimb coupling	
Utley et Sugden,1998 [50]	Cohort study	N = 11 Mean age (range) = 7.9 (5–12) Classification = uCP	3DMA	• Clinical scores: – • Quantitative measures: Mean, peak velocity, Intra/interlimb coupling, Duration of movement	
Utley et al. 2004 [51]	Cohort study	N = 8 Mean age (range) = 8.1 (5–11) Classification = uCP	3DMA	• Clinical scores: – • Quantitative measures: • Mean, peak velocity, Intra/interlimb coupling, Grasp aperture, Duration of movement	
Utley et al. 2007 [52]	Case control study	N = 9 (+ 7 TDC) Mean age (range) = 7.8 (5–12) Classification = uCP	3DMA	• Clinical scores: – • Quantitative measures: Mean, peak velocity, Intra/interlimb coupling, Mean maximum displacement	
Van Thiel et al. 2001 [53]	Cohort study	N = 5 Mean age (range) = 18 (15–20) Classification = uCP	3DMA	• Clinical scores: – • Quantitative measures: Movement time, Joint displacement, Dysfluency of the hand movement, Onset asynchrony, Variability of onset asynchrony, Peak asynchrony, Variability of peak asynchrony	
Volman et al. 2020 [47]	Cohort study	N = 12 Mean age (range) = 10.7 (8–14) Classification = uCP	Digitizer	• Clinical scores: – • Quantitative measures: Cycle duration, Amplitude and circularity, Smoothness, Relative phase	

*Same cohort

**Same cohort

– none; 3DMA: 3D motion analysis; APS: Arm Profile Score; CHEQ: Children’s Hand-Use Experience Questionnaire; CIMT: Constraint-Induced Movement Therapy; CP: Cerebral Palsy; EA: Error Augmentation; HABIT: Hand-Arm Bimanual Intensive Therapy; H-HABIT: Home Hand-Arm Bimanual Intensive Training; IOC: Index of curvature; JTTHF: Jebsen–Taylor Hand Function Test; MACS: Manual Ability Classification System; MAX: maximum angular value; ROM: Range of Motion; SMT: synchronized metronome training; SPARC: Spectral Arc length; TDC: typically developing children; uCP: unilateral Cerebral Palsy

### Type of instruments

The 31 studies used 3 different types of measurement systems: (1) 3DMA, including optoelectronic systems (Optotrack^®^, VICON^®^, Qualisis^®^, MIE Medical Research^®^) [24, 25, 27, 28, 30–36, 38–42, 44–46, 49–53] and electromagnetic systems (Polhemus^®^) [26, 29]), (2) accelerometers (ActiGraph^®^) [23, 26] and (3) other instruments (bespoke instrumented cube [43], Kinect device [48] or digitizer with two pens [47]) (Table 2). The 3DMA studies used models with 2 to 41 markers positioned on both ULs. Markers were always positioned on the wrists, they were also positioned on the trunk in 9 studies and on the hips in 1 study [44]. They used between 3 and 13 infrared cameras with a sampling frequency of 50–200 Hz. Few studies reported the biomechanical model used for the data analysis. The studies that used actimetry all used two accelerometers: one on each wrist and none on the trunk [23, 26].

**Table 2:** Detailed characteristics of the quantitative measurement systems

Articles	System	Device, Manufacturer	Type, Size	Markers (N)	Bilateral marker location	Cameras (N)	Frequency Sampling	Kinematic Model	Algorithm	Data processing	Filter
3D motion analysis											
Cacioppo et al. 2020 [24]	Optoelectronic	Infrared cameras (Vicon^®^ system, Oxford Metrics, UK)	Reflective markers (9 mm)	26	Trunk, arms, forearms and hands	10	100 Hz	Euler sequence ISB	Shoulder joint centre (functional method)	Matlab® (Mathworks, Natick, MA, USA)	–
Cope et al. 2010 [30]	Optoelectronic	Skill Technologies 3D Motion Capture and Analysis System (1202 E. Maryland Ave. Suite 1G, Phoenix, AZ)	Motion sensors	8	Upper limbs and trunk	–	120 Hz	–	–	–	A low pass filter (6 Hz)
Feltham et al. 2010a [31]	Optoelectronic	Two serially-connected units of 3 infrared cameras (3020 Optotrak^®^, Northern Digital Inc., Waterloo, Canada)	Light emitting diodes	2 × 3	Dorsal tuberculum of the radius	2 × 3	200 Hz	–	–	–	–
Gaillard et al. 2019 [25]	Optoelectronic	Infrared cameras (Optotrack, Motion Analysis, Corvallis, OR, USA)	Reflective markers (9 mm)	26 (on the child) + 4 (objects)	Trunk, arms, forearms and hands	12	100 Hz	Euler sequence ISB	Shoulder joint centre (functional method)	Matlab® (Mathworks, Natick, MA, USA)	–
Gordon et al. 2007 [26]	Electromagnetic	Electromagnetic motion tracking System, Polhemus Fastrack (Polhemus, Colchester, Vermont, USA)	Electromagnetic position sensors	2	Each wrist	–	60 Hz	–	–	–	–
Howcroft et al. 2012b [32]	Optoelectronic	Infrared cameras (Vicon^®^ system, Oxford Metrics, UK)	Reflective markers (14 mm)	16	Third MCP joint, medial and lateral wrist, lateral aspect of the forearm, medial and lateral condyles of the humerus, lateral aspect of the upper arm, left and right acromions, C7, and midpoint of the clavicles	7	60 Hz	Upper body model, 7 segments (shoulder girdle, left upper arm, right upper arm, left lower arm, right lower arm, left hand, and right hand)		Vicon Bodybuilder	A low pass filter (6 Hz)
Hung et al. 2004 [33] Hung et al. 2010 [34]	Optoelectronic	Infrared cameras (Motion Analysis Corporation, USA)	Reflective markers	2	On the midpoint of the bilateral wrists	4	120 Hz	–	–	Eva 5.36 (Motion Analysis Corporation)	A low pass filter (6 Hz)
Hung et al. 2011 [35]	Optoelectronic	Infrared cameras (Motion Analysis Corporation, USA)	Reflective markers	2	On the midpoint of both wrists	8	120 Hz	–	–	Vicon software	A low pass filter (6 Hz)
Hung et Meredith, 2014 [36]	Optoelectronic	Infrared cameras (Vicon^®^ system, Oxford Metrics, UK)	Reflective markers	41	Bilaterally on the anterior and posterior portions of the head, the shoulders (acromion process), the elbows (lateral epicondyle), the wrists (radio and ulnar styloid processes), the hands (index MCP joint), the upper arms, the forearms, between the clavicles, on the sternum, on C7, on T10, and on the right scapula		120 Hz	Whole-body plug-in-gait model		Vicon Nexus 1.51	A low pass filter (6 Hz)
Hung et al. 2017a. [27]	Optoelectronic	Infrared cameras (Vicon^®^ system, Oxford Metrics, UK)	Reflective markers	7	C7 and bilateral shoulder (acromion process), elbow (lateral epicondyle), and wrist (ulnar styloid process)	8	120 Hz	–	–	Vicon workstation 4.6	A low pass filter (6 Hz)
Hung et Spingarn, 2018 [37]	Optoelectronic	Infrared cameras (Vicon^®^ system, Oxford Metrics, UK)	Reflective markers	41	Bilaterally on the anterior and posterior portions of the head, the shoulders (acromion process), the elbows (lateral epicondyle), the wrists (radio and ulnar styloid processes), the hands (index MCP joint), the upper arms, the forearms, between the clavicles, on the sternum, on C7, on T10, and on the right scapula	8	120 Hz	Whole-body plug-in-gait model	–	Vicon Nexus 1.51	A low pass filter (6 Hz)
Hung et al. 2018 [38]	Optoelectronic	Infrared cameras (Vicon^®^ system, Oxford Metrics, UK)	Reflective markers	7	C7 and bilateral shoulder (acromion process), elbow (lateral epicondyle), and wrist (ulnar styloid process)	8	120 Hz	–	–	Workstation 4.6 (Vicon, Denver, CO, United States)	A low pass filter (6 Hz)
Hung et al. 2019 [28]	Optoelectronic	Infrared cameras (Vicon^®^ system, Oxford Metrics, UK)	Reflective markers	7	Bilateral shoulder (acromion process), elbow (lateral epicondyle), wrist (ulnar styloid process), and spinous process of C7	8	120 Hz	–	–	Workstation 4.6 (Vicon, Denver, CO, United States)	A low pass filter (6 Hz)
Hung et Zeng, 2020. [39]	Optoelectronic	Infrared cameras (Vicon^®^ system, Oxford Metrics, UK)	Reflective markers	9	Bilaterally on the shoulders (acromion process), the elbows (lateral epicondyle), the wrists (ulnar styloid processes), the hands (index MCP joint) and on C7	8	120 Hz	–	–	Vicon Nexus 1.51	A low pass filter (6 Hz)
Johansson et al. 2012 [40]	Optoelectronic	Infrared cameras (Proreflex, Qualisys Inc., Gothenburg, Sweden)	Reflective markers (7–29 mm)	9	Left and right shoulders, elbows, wrists and knuckles of index finger, and one forehead marker	6	120 Hz	–	–	Matlab® (The Mathworks Inc., Boston, MA)	A second-order 10 Hz dual pass Butterworth filter
Johansson et al. 2014 [41]	Optoelectronic	Infrared cameras (Proreflex, Qualisys Inc., Gothenburg, Sweden)	Reflective markers (12–29 mm)	6	Left and right shoulders, elbows, and wrists	6	120 Hz	–	–	Matlab® (The Mathworks Inc., Boston, MA, USA)	A second order 12 Hz Butterworth filter
Klotz et al. 2014 [42]	Optoelectronic	Infrared cameras (Vicon-M-series, Oxford Metrics, Oxford, UK)			The Heidelberg Upper Extremity model (HUX) + 3 on the head, and 1 twin-marker placed on the upper arm	12	120 Hz	Heidelberg Upper Extremity model (HUX)	Joint centres & joint axes (functional methods)	–	–
Rudisch, et al. 2016 [29]	Electromagnetic	Electromagnetic motion tracking System, Polhemus^®^ G4 (Polhemus, Colchester, Vermont, USA)	Electromagnetic position sensors	2	Dorsally over the 3rd metacarpal bone		120 Hz	–	Euler Angles allowing the projection of the centre of measurement into the centre of the hand A semi-automated algorithm	-Customized software, written in labview 2014 (National Instruments, Austin, Texas, USA) -MATLAB® R2014b (The Mathworks Inc., Natick, MA, USA)	–
Smorenburg et al. 2011 [44]	Optoelectronic	Two serially connected units of 3 infrared cameras (3020 Optotrak^®^, Northern Digital Inc., Waterloo, Canada)	Light emitting diodes	8	Dorsal tuberculum of the radius (wrist), lateral epicondyle of the humerus (elbow), greater tubercle of the humerus (shoulder) and the trochanter of the femur (hip)	2 × 3	200 Hz	–	–	–	–
Smorenburg et al. 2012a [45]	Optoelectronic	One unit with 3 infrared cameras (3020 Optotrak^®^, Northern Digital Inc., Waterloo, Canada)	–	–	–	3	200 Hz	–	–	Custom-made Matlab programmes (The Mathworks, version 7.1)	–
Smorenburg et al. 2013 [46]	Optoelectronic	One unit with 3 infrared cameras (3020 Optotrak^®^ Northern Digital Inc., Waterloo, Canada)	Light emitting diodes	–	–	3	200 Hz	–	–	Custom-written Matlab routines (The Mathworks, version 2011)	–
Steenbergen et al. 2008 [47]	Electromagnetic	Electromagnetic motion tracking System (Pohlemus^®^ Fastrack, Colchester)	Electromagnetic position sensors	–	–	–	400 and 60 Hz	–	–	SC/ZOOM, Umeå University, Sweden	–
Sugden et Utley, 1995 [49]	–	–		38	19 markers on each arm: Shoulder, elbow, wrist (head of the radius and ulna), fingers (the joint of the metacarpals and proximal phalanges, the joint of the proximal phalanges and the middle phalange and the tip of the distal phalanges), thumb (the joint of the carpals and metacarpals, the joint of the metacarpals and the proximal phalanges; and the tip of the distal phalanx)	–	Digitised at 50 Hz for a range of 20 to 80 frames	–	–	–	–
Utley et Sugden,1998 [50]	Optoelectronic	3 CCD cameras with coaxial infrared amys (MIE Medical Research Ltd, Leeds. UK)	Luminous lightweight spheres (1 cm)	8	Bilaterally shoulder, elbow, wrist, and middle digit	3	–	Calibration frame with nine control points (50 cm^3^)	Sealing method (direct linear parameters calculated)	IHM-compatible interface card in an Elonex PC-i33 computer with appropriate sofnvare	–
Utley et al. 2004 [51]	–	–	Luminous lightweight spheres (1 cm)	10	Bilaterally shoulder, elbow, wrist, first digit and thumb	3 + 1 for the video	50 Hz	–	–	–	–
Utley et al. 2007 [52]	Optoelectronic	Kinematrix optoelectronic recording system (MIE Medical Research Ltd, Leeds, UK) )	Reflective markers (1 cm)	4	Wrists and middle digit of each hand	3	50 Hz	Calibration frame with nine control points (50 cm^3^)	–	–	–
Van Thiel et al. 2001 [53]	Optoelectronic	Infrared cameras (3020 Optotrak^®^, Northern Digital Inc., Waterloo, Canada)	Light Emitting Diodes	4 or 5 (hitting task)	Wrist and shoulder of both arms Hitting task: shoulders + 3 attached to the end of the rod		200 Hz	–	Absolute euclidean distance (shoulder)	–	A second order Butterworth filter of 20 Hz
Accelerometers											
Beani et al. 2020 [23]	Accelerometers	ActiGraph GT3X + monitor (wGT3X-BT Monitor, ActiGraph, Florida, FL, model 7164; 4.6 × 3.3 × 1.5 cm, 19 g)	Activity monitor	2	Each wrist		80 Hz	–	Data were recorded in 3 axes and downloaded using ActiLife v.6.13.3 software (ActiGraph, Pensacola, FL)	–	–
Gordon et al. 2007 [26]	Accelerometers	Manufacturing Technology Inc. Fort Walton Beach, FL, model 7164; 5.1 × 2.6 × 1.5 cm, 42.9 g	Activity monitor	2	Each wrist	–	10 Hz	–	Activity counts	–	–
Other instruments											
Mutalib et al. 2019a. [43]	A bespoke instrumented cube (10 × 10 × 10cm; 530 g)	Each face of the cube was equiped with a force transducer (TAL107F, HT Sensor Technology Co., Ltd) - A 9-degree of freedom Bosch BNO055 Inertial Measurement Units (IMU) inside the cube		–	–	–	Recorded wirelessly over Bluetooth at 60 Hz and 10-bit resolution	–	–	- A custom data collection program running on an Android tablet, created in the Unity game engine (Unity Technologies, USA) - Matlab^®^ (The MathWorks, USA)	–
Shum et al. 2020 [48]		Kinect v2 (Microsoft Corporation, Redmond, USA)	–	–	–	–	Recorded at 90 Hz, the Oculus system’s inherent sampling frequency, and resampled at 30 Hz prior to further calculation	–	–	–	–
Volman et al. 2020 [47]	Digitizer	A digitizer (Wacom Intuos A3) and two cordless pens (Intuos GP 300) with a computer	–	–	–	–	100 Hz; spatial accuracy 0.25 mm	–	–	OASIS software package	Low pass filtered (Butterworth dual pass, cut-off frequency10 Hz)

– none; Hz: Hertz; IMU: Inertial Measurement Unit; ISB: International Society of Biomechanics; MCP: metacarpophalangeal

Most of the protocols assessed functional tasks (Table 3). This included daily tasks (ex: placing a hat on the head, opening a drawer, picking up a box, food preparation, playing, etc.) performed in a laboratory setting (n = 13), that were mainly recorded with 3DMA systems [26–30, 33–38, 42, 48]. Child-friendly protocols were used, including game scenarios (n = 5), such as 3D virtual environment [48], videogames (Kinect [48], Nintendo Wii [32]) or projection of a scene on a screen [24, 25, 53]. The drawer opening task was the most frequently used (n = 7) [26–28, 33–35, 38], followed by bimanual reach to grasp tasks (n = 5) [36, 43, 48–52], a box pick-up task (n = 3) [29, 36, 37] and bimanual circular movements (n = 3) [31, 44, 47]. The studies that used actimetry recorded the Assisting hand assessment (AHA) test session, rating the effectiveness of affected hand use during semi-structured, bimanual play activities [23, 26]. No studies evaluated spontaneous bimanual movement during free play or activities of daily living and none evaluated movement in the child’s home environment. Ten studies explored a combination of bimanual tasks (2–5 different tasks). In 5 studies, data from the bimanual tasks were only analysed for the impaired UL and not both ULs [24, 25, 30, 42, 48].

**Table 3:** Description of protocols and tasks for bimanual assessment

Articles	Setting	Material	Task description	Starting position	Condition	Velocity condition	Practice trials	Number of trials	Protocol duration	Number of sessions	UL analysed
Beani et al. 2020 [23]	Clinical environment	AHA set up	A session of semi-structured playing activity (AHA session)							1 session	Both UL
Cacioppo et al. 2020 [24]	Laboratory	A game set-up (airplane cockpit): 2-handed joystick, turbo, shifter, dashboard, box and buzzer	A five-task protocol, Be An Airplane Pilot 2.0: "flying over mountains", "slaloming", "hooking the luggages", "opening the door", "refueling"	Upright sitting on an adjustable chair with 90°of hip, knee and elbow flexion. The forearm and hand were positioned on the table	1 condition: movement of interest performed by the impaired UL	At a self-selected speed	1 practice trial	5 trials except task 5 (4 trials)	30 min	2 sessions (2–4 weeks)	Impaired UL
Cope et al. 2010 [30]			To place a hat on the head			At a self-selected speed				Pre, post therapy, at 3- and 6-month follow ups (3 uCP)	Impaired UL
Feltham et al. 2010a [31]	Laboratory	2 arm ergometers (871E, Monark Exercise AB, Vansbro,Sweden), each with a handle, attached to the edge of a wooden disc such that it spun freely through 360° around a vertical axis fixed to a wooden table top	An inward, symmetrical circular movement of both upper limbs and maintain this coordination mode throughout the experiment (to rotate the discs continuously)	Sitting on a height-adjustable stool and placed one UL on either side of the divide and angled their head toward the side of their dominant/ less impaired UL, both feet flat on the floor, knees flexed to 90° and elbows flexed to 90°. The ULwere at the inner most part of each of circle	3 conditions according to the divide placed between the arms	At a self-selected speed	Yes (no number)	3/condition	Each trial = 15 s		Both UL
Gaillard et al. 2019 [25]	Laboratory	A game set-up (airplane cockpit) (2-handed joystick, turbo, box and 2 buzzers)	‘‘Be an Airplane Pilot’’ (BE-API protocol) 4 bimanual tasks: ‘‘mountain passing’’, ‘‘slaloming’’, ‘‘dropping parachutists’’, ‘‘refueling’’	Sitting on an adjustable chair with 90° of hip, knee and elbow flexion. The forearm and hand were positioned on the table	1 condition: movement of interest performed by the impaired UL	At a self-selected speed	1 practice trial	4 cycles of movement except task 4 (3 cycles of mvt)	1 h	1 session = 3 trials (1 trial = 4 tasks) (inter trials)	Impaired UL
Gordon et al. 2007 [26]	Laboratory	1) AHA set up 2) The drawer hand a loop handle (9 × 3 cm) and was placed in front of the participant at midline 30 cm from the edge of the table; A 14 × 10 cm push-button light switch	1) AHA testing session (accelerometers) (12 activities performed) 2) a drawer-opening task*	Sitting 15 cm in front of a table with their elbows flexed at right angles with their hands palm down on the edge of the table, 30 cm apart	2 conditions: with each hand opening the drawer	At a self-selected speed		5 trials × 2 (10 trials)		Before, within the first week after and at 1-month post-intervention	Both UL
Howcroft et al. 2012b [32]	Laboratory	Nintendo Wii, “Wii Sport “ and “Dance Dance Revolution “ games, Wii remote, nunchuck and dance mat	2/4 AVGs: bowling (bilateral) and a dance game (quadrilateral)		Played each game in a randomized order on a preselected beginner level		Familiarize with the games for a maximum of 5 min before playing		Each game for 8 min with a rest period of 5 min between each game	1 session	Both UL
Hung et a., 2004 [33]	Laboratory	The drawer (15 × 15 cm) with loop handle placed in front of the subject at midline 30 cm from the edge of the table a push-button light switch	A drawer-opening task*	Sitting 15 cm in front of a table with their elbows flexed at right angles; hands 30 cm apart and slightly closed with the palm facing down at the edge of the table	4 conditions: * with each hand opening the drawer *speed (self-paced vs fast-as-possible)	At a self-selected speed and fast-as-possible speed	3 practice trials	5 trials/ condition (total of 20)		1 session	Both UL
Hung et al. 2010 [34]	Laboratory	The drawer (15 × 9 × 15 cm) placed in front of the subject at midline 30 cm from the edge of the table + an exchangeable handle, either a loop or a knob attached to the front of the drawer + either a ‘‘large’’ (14 × 9 × 10 cm) or a ‘‘small’’ (1.5 × 9 × 2 cm) push-button light switch placed inside the drawer	A drawer-opening task*	Sitting 15 cm in front of the table with their elbows flexed at right angles and hands positioned 30 cm apart at the edge of the table	8 conditions: two handles (knob, loop), two switches (small, large), each hand	At a fast-as possible speed	3 practice trials	5 trials/condition		1 session	Both UL
Hung et al. 2011 [35]	Laboratory	The drawer (15 × 15 cm) with a loop handle (9 × 3 cm) was placed at midline 30 cm from the edge of the table A push-button light switch (14 × 10 cm)	A drawer-opening task*	Sitting 15 cm in front of the table with their elbows flexed at right angles and hands positioned 30 cm apart at the edge of the table	1 condition: to open the drawer with the less affected hand and to insert the more affected hand in the drawer	At a self-selected speed				Before and after intensive practice (HABIT/CIMT)	Both UL
Hung et Meredith, 2014 [36]	Laboratory	An empty plastic box (weight: 0.6 kg, length: 0.45 m, width: 0.29 m, height: 0.17 m)	A simple box pick-up task (dual task condition): to reach down, grasp, and pick up an empty box to waist height without touching their body	Standing	3 conditions: standing, walking while carrying nothing (baseline condition) and walking while carrying the same empty plastic box with two hands (dual task condition)	At a self-selected speed	3 practice trials	5 trials × 2 conditions		1 session	Both UL
Hung et al. 2017a. [27]	Laboratory	The drawer (15 × 15 cm) with loop handle placed in front of the subject at midline 30 cm from the edge of the table. A push-button light switch (14 × 10 cm)	A drawer-opening task*	Sitting 15 cm in front of the table with their elbows flexed at right angles and hands positioned 30 cm apart at the edge of the table		At a self-selected speed	2 practice trials	5 trials		Before and after intensive practice	Both UL
Hung et Spingarn, 2018 [37]	Laboratory	Empty plastic box (length: 45 cm, width: 29 cm, height: 17 cm) one inch in front of their toes to waist height	A simple box pick-up task (dual task condition): to reach down, grasp, and pick up an empty box to waist height without touching their body	Sitting quietly with their feet separated about shoulder width		At a self-selected pace	2 practice trials	5 trials		1 session	Both UL
Hung et al. 2018 [38]	Laboratory	The drawer (15 × 15 cm) with loop handle placed in front of the subject at midline 30 cm from the edge of the table. A push-button light switch (14 × 10 cm)	A drawer-opening task*	Sitting 15 cm in front of the table with their elbows flexed at right angles and hands positioned 30 cm apart at the edge of the table		At a self-selected speed	2 practice trials	5 trials		Before, immediately after and 6 months after training	Both UL
Hung et al. 2019 [28]	Laboratory	The drawer (15 × 15 cm) with loop handle placed in front of the subject at midline 30 cm from the edge of the table. A push-button light switch (14 × 10 cm)	A drawer-opening task*	Sitting 15 cm in front of the table with their elbows flexed at right angles and hands positioned 30 cm apart at the edge of the table		At a self-selected speed	2 practice trials	5 trials		1 session	Both UL
Hung et Zeng, 2020. [39]	Laboratory	Two sizes of the tray handle (small: 2.54 cm; large: 3.81 cm) and the water bottle (80 ml, 3.5 × 11 cm)	To lift a tray with a water bottle on top: participants reached forward, grasped, and lifted the tray up with both hands at about 8 cm from the table top leveled and then counted from 1 to 5 before they put it down	Sitting 15 cm in front of a table with elbows flexed at right angle, and hands slightly closed located at the edge of the table. The tray was positioned in the middle of the table	4 conditions: 2 handle size and cap condition (with/without)	At a self-selected speed	2 practice trials	5 trials		1 session	Both UL
Johansson et al. 2012 [40]	Laboratory	Test platform with 10 integrated easy to press light-switches	To begin from a starting point, pressing three light-switch buttons in a sequential order (both ULs simultaneously)	Sitting in a chair in front of the custom-made test platform	4 directions (extension, flexion, adduction, abduction)		2 practice trials/ condition	3 usable trials/ direction (12)		3 sessions (pre, post and at 6 months post training)	Both UL
Johansson et al. 2014 [41]	Laboratory	Test platform with 10 integrated light-switches	To begin from a starting point, pressing three light-switch buttons in a sequential order (both ULs simultaneously)		4 directions (extension, flexion, adduction, abduction)			12 trials		3 sessions (pre, post and at 6 months post training)	Both UL
Klotz et al. 2014 [42]	Laboratory	(1) Two cups (2) A paperboard box	(1) Decanting cups: to hold two cups, one in each hand and to decant the cereals into the empty cup (2) Moving a box on a desk: this box had to be pulled towards, turned 180° around and then thrown/shoved over the table-edge	Sitting on a chair with adjustable sitting position. In front of the person a paperboard box was placed		At a self-selected speed		Decanting cups × 6 trials (× 3 for each body side) Moving a box on a desk: × 3 trials		1 session	Impaired UL
Mutalib et al. 2019a. [43]	Laboratory	Bespoke instrumented cube (10 × 10 × 10 cm; weight = 530 g)	Single-object bimanual lifting task: to grasp the cube with both hands, move it vertically for approximately 8–10 cm, hold for 1–2 s, and then return back to the start position (lift-static-deposit)	Sitting on a chair with adjustable sitting position, in front of a table where the cube was placed		At a self-selected speed	The first 5 trials	15 trials with a 10-s interval between trials		1 session	Both UL
Rudisch, et al. 2016 [29]	Laboratory	The box was positioned at a distance of 25 cm from the edge of the table and adhered to the table	A box-opening task: to open a box with their affected- or less-affected hand and pressing a button inside with the opposite hand	Sitting on a height adjustable chair with both feet touching the floor (or footplate), in front of a height adjustable table, with elbows flexed at a 90° angle with hands resting on the table surface	2 conditions: the affected hand or less affected handopening the box	At a self-paced, comfortable speed		10 times; 5 x / condition;	5 min for set up + 5 min to administer the task	1 session	Both UL
Shum et al. 2020 [48]	Laboratory 2 × 2 m physical “play-space”	The Oculus Rift system (Oculus VR, LLC, Menlo Park, CA, USA), the Oculus Touch controller pair, and two Oculus Sensors; within a 3D virtual environment developed in Unity 3D 5.0 2017 (Unity Technologies, San Francisco, USA)	A reaching task: to pick up and move the virtual objects to a specified location with both hands simultaneously. Items of food preparation (hotdog onto a bun, meat into a dumpling, rice onto nori, and shrimp into a sushi roll)	Sitting on a chair, Oculus Sensors and Kinect v2 placed 1.5 m from the play-space origin to maximize the field of view	2 conditions; with or without visual error augmentation The objects were randomly varied at every 5th, 7th, and 8th trial to mitigate boredom		At least 3 practice trials	One set = baseline + training trials (60 training + 5 evaluation trials) + wash out (15 trials)		1 session, 2 sets	Impaired UL
Smorenburg et al. 2011 [44]	Laboratory	A glass, opaque screen or mirror divide, between the arms along the midsagittal plane. Handle was attached to a wooden disc which spun freely 360° around a vertical axis. The axes fixed to a wooden plateau and located 0.31 m apart	A bimanual symmetrical circular movement: to perform a continuous inward symmetrical circular bimanual movement (the right UL rotated CCW and the left UL rotated CW). Children were asked to rotate the discs continuously to keep the movement time per cycle	Sitting on a height adjustable chair at a height adjustable table with the knees flexed to 90°	-3 visual feedback: (1) the participant viewed both ULs, (2) only one arm and (3) one UL and its mirror reflection -2 head orientation conditions: looking from the impaired and from the less-impaired body side	At a self-selected speed		3 trials/ condition	Each trial = 15 s	1 session	Both UL
Smorenburg et al. 2012a [45]	Laboratory	A custom-made wooden construction consisting of two handles on two separate parallel tracks 20 cm apart. direction Handles could be moved anterior–posterior	A bimanual matching task: to match the position of a target with both arms at the same time i.e., to move both hands towards the target as symmetrically as possible starting with the handles at the beginning of the track, i.e., 0% MRD	Sitting on a height adjustable chair at a height adjustable table with the knees flexed to 90°	2 conditions -4 target positions: 25%, 50%, 65%, or 80% of the MRD (less-impaired hand side) -2 visual conditions: opaque screen and mirror		1 practice trial	2 trials per condition (16 trials in total)		1 session	Both UL
Smorenburg et al. 2013 [46]	Laboratory	A custom-made wooden box with two handles in a slit, one at each side of an opaque divide, running parallel in the sagittal and horizontal plane. The handles were located 20 cm apart and the maximum anterior–posterior range was 56 cm. The handles inside the box were attached to two handles outside the box on which light emitting diodes were attached	A bimanual matching task: to move the two handles to the target with the impaired and the less-impaired arm simultaneously and in a symmetrical fashion	Sitting on a height adjustable chair behind a height adjustable table with the knees flexed to 90°	1 condition: *Target located at 20%, 40%, 60%, 70%, and 80% MRD			2 trials per target position (10 trials)		3 sessions: pre, post (immediately), and after a 1-week-retention	Both UL
Sugden et Utley, 1995 [49]	Laboratory	Small half-spheres (1) and cubes (2,3)	**(1)** to reach for and touch two targets placed directly in front of them. **(2)** reached for and grasped cubes placed directly in front of them. **(3)** a double movement: the child had to reach to touch a marked box 45" to hislher side and then reach for and grasp the cube	(1) sitting on his/her usual chair at table, feet on the ground or a fixed surface. (2) (3): sitting on trip trap, hands placed in a standardised starting position	1 condition: both hands moving simultaneously	At a self-selected speed	3 practice trials	3 trials		1 session	Both UL
Utley et Sugden,1998. [50]	Laboratory	The target object: a small piece of card or wooden cube. The cube size (3, 4, 5 cm) and distance travelled (20, 25, 30 cm) according to the size of the child	3 reaching tasks: (1) reach and touch, (2) reach and grasp, (3) reach, touch, and grasp	Sitting at an adjustable desk in a Tripp Trapp, feets in contact with the ground or the footplate on the chair	1 condition: both hands moving simultaneously		3 practice trials	3 trials	20–30 min	1 session	Both UL
Utley et al. 2004 [51]	Laboratory	2 wooden cubes (small and large). The cubes were placed 20, 25 or 30 cm away from the subject in the sagittal direction, according to the size of the child	To reach and grasp bimanually to a small cube (1.5 cm) and a large cube (6 cm)	Sitting at an adjustable desk and chair	*small and large cube *order of unimanual and cube's size alternatively chosen	At a self-selected speed	3 practice trials	3 trials bimanually	20–30 min	1 session	Both UL
Utley et al. 2007 [52]	Laboratory	The cube size (3, 4, 5 cm) and distance traveled (20, 25, 30 cm) were varied according to the size of the child	To pick up (reach and grasp) a cube bimanually when the surface it was placed on was either sloping away from the child (Experiment 1) or towards the child (Experiment 2)	Sitting at an adjustable desk in a Tripp Trapp, feets in contact with the ground or the footplate on the chair	2 conditions: the surface the cube was placed on was either sloping away from the child or towards the child		3 practice trials	3 trials /condition		1 session	Both UL
Van Thiel et al. 2001 [53]	Laboratory	Hitting task (H): a rod (21 cm; diam 2,5 cm) in each hand; attached to the tip of rod was the pod of a badminton shuttle cock to enable firm but safe impacts with the screen H: targets on a screen, diameter 2 cm (small) and 5 cm (large); Reaching task (R): buttons of 3 and 5 cm in diameter Grasping task (G): blocks width of 3 and 5 cm	H: to hit target with both hands; with the tip of the rod quickly, immediately after the target appearance R: to push a button G: grasp and lift small blocks / to hit the target	Sitting at a table and resting her/his hand on start boxes	2 conditions: target sizes (small/large)	As quickly as possible		12 trials / condition (= 24 per task)		1 session	Both UL
Volman et al. 2020 [47]	Laboratory	A template with two circles (diameter: 9 cm; distance between the circle centres: 23 cm) was placed on the digitizer under a transparent overlay	To hold the pen––as far as possible––with a power grip of all fingers and to do bimanual (asymmetric and symmetric) circle drawing movements (15 circles)	Sitting at a table in a Tripp Trapp chair	4 conditions: (1) symmetric bimanual inward (left CW and right CCW); (2) symmetric bimanual outward (left CCW and right CW); (3) asymmetric bimanual CW (both hands CW); (4) asymmetric bimanual CCW (both hands CCW)	At a self-selected speed, instructed to move, as smooth as possible, and not to stop moving	3 practice trials	3 trials/ condition (totally: 12)	30 min	1 session	Both UL

*To open a spring-loaded drawer with one hand and to insert the contralateral hand in the drawer to activate a push-button light switch

Assisting Hand Assessment (AHA); Active video games (AVG); clockwise (CW); counterclockwise (CCW); Upper Limb (UL)

In most studies, the starting position was almost the same: sitting on a chair at a table with the knees and elbows flexed at 90° [24–29, 31, 33–35, 37–40, 42–53]. The tasks were performed under different conditions: object size, height and grasp-type [34, 39, 51, 53], target position [40, 41, 45, 46, 52], environmental feedback (ex: mirror or opaque screen) [31, 44–46], each hand performance of asymmetrical bimanual tasks [26, 29, 33, 35, 47], or increasing difficulty [32]. Participants performed tasks at their self-selected speed, and 3 studies analysed maximum speed [33, 34, 53]. One to 5 trials were recorded, with at least 1 practice trial, except for the accelerometery measures that were recorded directly [23, 26]. The children and adolescents performed between 1 and 4 sessions of each protocol (most often 1 session [23, 25, 28, 29, 32–34, 36, 37, 39, 42–45, 47–53]. Protocol durations were from a few minutes to 1 h.

### Outcome measures

Instrumented measurements were mainly used to calculate spatiotemporal variables during bimanual movements (26/32 studies) [27–35, 37–44, 48] *(Table **4**)*. Movement time, mean and peak velocity were the most frequently measured. Trunk and upper limb joint (mostly elbow and wrist) kinematics were evaluated in 15 studies [24, 25, 27, 27, 28, 30, 32, 34, 38, 39, 42, 48, 51–53]. One study focused on hand movements [36]. Studies that evaluated movement quality analysed smoothness, trajectory (Index of Curvature (IOC) [24], accuracy [29] and circularity [47]. Smoothness was analysed by the number of movement units [30, 40, 41], spectral arc length (SPARC) [24, 43], number of velocity peaks [47, 48], normalized jerk [31], relative phase [45], and dysfluency of hand movement [53]. Four variables were calculated from accelerometery: mean activity count [23], asymmetry index [23], duration of limb use [26] and goal synchronization [26]. Bilateral movements were analysed with 6 specifically developed spatiotemporal variables: task completion time, goal synchronization of the hands, movement overlap time [27, 28, 33–35, 38], interlimb coupling [29, 31, 49–52], continuous relative phase [31, 44, 47] and asynchrony [53]. Task completion time, goal synchronization of the hands and movement overlap time were only analysed in the drawer-opening task [27, 28, 33–35, 38].

**Table 4:** Outcome measures evaluated in bimanual assessments

	Outcome measures				
	Spatio temporal	Kinematics	Quality	Actimetry	Others
Unilateral variables (= one hand analysed)	**Spatial** - 3D Distance (mm) [40, 41] - Location of peak velocity (frames) [49]	- Range of Motion Upper arm (°) [27, 38, 39, 48] Trunk (°) [25, 26, 42] Shoulder (°) [24, 25, 32, 37, 53] Elbow (°) [24, 25, 27, 30, 32, 36–39, 42] Wrist [24, 25, 30, 32] Vertical hand (mm) [36] Lateral hand (mm) [36] C7 displacement (mm) [27, 37–39] - Maximum angular value (°) [24, 25, 52] - Arm Profile Score (°) [24] - Angular waveforms (°) [25] - Difference in vertical position between hands (m) [36] - Grasp aperture (cm) [51]	**Smoothness** - SPARC [24, 43] - Number of movement units [30, 40, 41] - Normalized jerk [31] - Number of velocity peaks [47, 48] - Relative movement smoothness (peaks/cm) [45] - Dysfluency of the hand movement (velocity inversions/sec) [53]	- Mean activity count (activity count/sec) [23] - Asymmetry index [23] - Time of use (sec) [26] - Goal synchronization (sec) [26]	- Root Mean Square Error [49] - Absolute error [45, 46] - Hand height position differences (mm) [39]
	**Temporal** - Movement time / duration of movement (sec) [29–31, 37, 40, 42, 43, 47, 49–51, 53] - Time to peak velocity (sec) [48] - Two hands offset/ onset difference (sec) [37] - Timing differences at offset (sec) [39] - Intralimb coupling [50–52]				
			- Index of curvature [24] - Spatial accuracy (m) [29] - Amplitude (cm) and circularity [47]		
	**Velocity** - Peak velocity (mm/sec or m/sec) [30,39, 48,49–52] - Angular velocities (°/sec) [32] - Accelerations (°/sec2) [32] - Tangential velocity (mm/sec) [33, 34] - Peak velocity difference (mm/sec) [33, 35] - Average velocity (mm/sec) [45, 49–52]				
Bilateral variables (= both hands analysed)	- Task completion time (sec) [27, 28, 33–35, 38] - Goal synchronization (sec) [27, 28, 33–35, 38] - Movement overlap (%time) [27, 28, 33–35, 37] - Interlimb coupling [29, 31, 50–53] - Continuous relative phase (i.e. synchronicity) (°) [31, 44, 47] - Onset asynchrony (sec) [53] - Variability of onset asynchrony (sec) [53] - Peak asynchrony (sec) [53] - Variability of peak asynchrony (sec) [53]				
Total	22	6	9	4	4

IOC: Index of curvature; SPARC: Spectral Arc length

Outcome measures with no specified units are unitless measures

### Validation of metrological properties

In total, 22 studies evaluated one or more metrological properties of their instrumented measures [23–31, 33–43, 46, 48] (Table 5).

**Table 5:** Metrological properties assessed in the included studies

Articles	Measurement Tool	Task	Variable	Convergent validity (COSMIN score)	Discriminative validity (COSMIN score)	Interrater reliability	Intrarater reliability	Test–Retest	Responsiveness (COSMIN score)
						(COSMIN score)			
Beani et al. 2020 [23]	Accelerometers	AHA session	Mean activity count Asymmetry index	✓ MACS ✓ (7/12)	✓ TDC ✓ (8/9)				
Cacioppo et al. 2020 [24]	3DMA	BE API 2.0	ROM MAX APS SPARC IOC	✓ AHA ✓ (12/12)	✓ TDC ✓ (9/9)	✓ ICC, ✓ MDC ✓ (15/21)		✓ ICC, ✓ MDC ✓ (15/21)	
Cope et al. 2010 [30]	3DMA	To place a hat on the head	Peak velocity Movement time Movement units ROM						✓ CIMT ✓ (9/9)
Feltham et al. 2010a [31]	3DMA	Symmetrical circular movements	Duration Interlimb coupling Continuous relative phase Normalized jerk		✓ TDC ✓ (9/9)				
Gaillard et al. 2019 [25]	3DMA	BE API 1	Angular waveforms ROM MAX	✓ AHA, ABILAND-Kids (12/12)	✓ TDC ✓ (9/9)	✓ CMC ✓ ICC, SEM (15/21)	✓ CMC ✓ ICC, SEM (15/21)		
Gordon et al. 2007 [26]	Accelerometers; 3DMA	AHA session; a drawer-opening task	Time of use Goal synchrony		✓ control ✓ group (9/9)				✓ HABIT ✓ (9/9)
Howcroft et al. 2012b [32]	3DMA	Wii games	ROM Angular velocities Accelerations						
Hung et al. 2004 [33]	3DMA	A drawer-opening task	Task completion time Goal synchronization Movement overlap time Tangential velocity Peak velocity difference	✓ JTHFT (8/12)	✓ TDC ✓ (8/9)				
Hung et al. 2010 [34]	3DMA	A drawer-opening task	Tangential velocity Task completion time Goal synchronization Movement overlap Task hand movement time		✓ TDC ✓ (8/9)				
Hung et al. 2011 [35]	3DMA	A drawer-opening task	Peak tangential velocity Movement overlap time Goal synchronization Task completion time	*AHA ✓ (12/12)	✓ Control ✓ group ✓ (9/9)				✓ HABIT/ ✓ CIMT ✓ (9/9)
Hung et Meredith, 2014 [36]	3DMA	To hold an empty plastic box	Difference in vertical position between hands Vertical hand ROM Lateral hand ROM Elbow excursion Shoulder excursion		✓ TDC (9/9)				
Hung et al. 2017a. [27]	3DMA	A drawer-opening task	Movement overlap time Goal synchronization Task completion time C7 displacement Upper arm and elbow joint angle excursion		✓ Control ✓ group ✓ (9/9)				✓ Structured ✓/unstructured ✓ practice ✓ (9/9)
Hung et Spingarn, 2018 [37]	3DMA	A drawer-opening task	Movement overlap time Goal synchronization Task completion time C7 displacement Upper arm and elbow joint angle excursion						✓ H- ✓ HABIT ✓ (9/9)
Hung et al. 2018 [38]	3DMA	A box pick-up task	Movement time Two hands offset/onset difference Elbow, shoulder joint excursion		✓ TDC ✓ (8/9)				
Hung et al. 2019 [28]	3DMA	A drawer-opening task	Total movement time Goal synchronization Normalized movement overlap C7 displacement	✓ * AHA, ✓ MRI ✓ (12/12)					
Hung et Zeng, 2020. [39]	3DMA	To lift a tray with a water bottle	Peak velocity Upper arm and elbow joint C7 displacement Hand height position differences Timing differences at offset		✓ TDC, ✓ adults ✓ (9/9)				
Johansson et al. 2012 [40]	3DMA	Pressing 3 light-switch buttons	Duration of the movement 3D Distance Number of movement units						✓ SMT ✓ (5/9)
Johansson et al. 2014 [41]	3DMA	Pressing 3 light-switch buttons	3D Distance Number of movement units						✓ SMT ✓ (7/9)
Klotz et al. 2014 [42]	3DMA	Decanting cup and moving a box on a desk	Movement time ROM	✓ MACS & ✓ *ABILHAND-Kids (12/12)	✓ TDC ✓ (9/9)				
Mutalib et al. 2019a. [43]	Bespoke instrumented cube	Lifting task	Duration Isometric grasp force Interlimb force asymmetry SPARC		✓ TDC ✓ (9/9)				
Rudisch, et al. 2016 [29]	3DMA	Box-button task	Total task duration Temporal Coupling Spatial accuracy	✓ CHEQ & ✓ JTHFT ✓ (9/12)					
Shum et al. 2020 [48]	Kinect	A reaching task	ROM, RMSE Peak velocity Time to peak velocity Smoothness Trunk compensation	✓ MACS, (5/12)	✓ TDC (3/9)				✓ With/without ✓ EA ✓ (7/9)
Smorenburg et al. 2011 [44]	3DMA	Bimanual circular movement	Continuous relative phase						
Smorenburg et al. 2012a [45]	3DMA	A bimanual matching task	Absolute error Average velocity Relative movement smoothness						
Smorenburg et al. 2013 [46]	3DMA	A bimanual matching task	Absolute error						✓ Visual feedback/mirror feedback (9/9)
Sugden et Utley, 1995 [49]	3DMA	Reach, grasp small spheres and cubes	Duration Mean, peak velocity Location of peak velocity Interlimb coupling						
Utley et Sugden,1998. [50]	3DMA	Reaching tasks	Mean, peak velocity Intra/interlimb coupling Duration of movement						
Utley et al. 2004 [51]	3DMA	Reaching grasping tasks	Mean, peak velocity Inter/intralimb coupling Grasp aperture Duration of movement						
Utley et al. 2007 [52]	3DMA	To pick up a cube	Mean, peak velocity Inter/intralimb coupling Mean maximum displacement						
Van Thiel et al. 2001 [53]	3DMA	Hitting, reaching and grasping tasks	Movement time Joint displacement Dysfluency of the hand movement Onset asynchrony Variability of onset asynchrony Peak asynchrony Variability of peak asynchrony						
Volman et al. 2020 [47]	A digitizer and two cordless pens	Circle drawing movements	Cycle duration Amplitude and circularity Smoothness Relative phase						

*No correlation was found

The COSMIN score risk of bias checklist 2017 (Mokkink et al. 2020) was used to calculate a quality score for each metrological property. Each item in the boxes was rated as very good (3 points), adequate (2 points), doubtful (1 point) or inadequate (0 points). Convergent validity was assessed with Box 9.a (score/12), discriminative validity with Box 9.b (score/9), reliability with Box 6 (score/9) and responsiveness with Box 10.a (score/9)

✓ Variables for which the metrological property was assessed

3DMA: 3D motion analysis; APS: Arm Profile Score; CHEQ: Children’s Hand-Use Experience Questionnaire; CIMT: Constraint-Induced Movement Therapy; CMC: Coefficient of Multiple Correlation; EA: Error Augmentation; HABIT: Hand-Arm Bimanual Intensive Therapy; H-HABIT: Home Hand-Arm Bimanual Intensive Training; ICC: Intra Correlation Class; IOC: Index of curvature; JTTHF: Jebsen–Taylor Hand Function Test; MDC: Minimum Detectable Change; MRI: Magnetic Resonance Imagery; MAX: maximum angular value; RMSE: Root-Mean-Squared Error; ROM: Range of Motion; SMT: synchronized metronome training; SPARC: Spectral Arc length

#### Convergent validity

The mean COSMIN score of the studies that assessed convergent validity was 10/12 points.

Seven of the 31 studies evaluated correlations between the variables calculated from the instrumented assessments and scores on clinical assessments. Of these 7 studies, 3 used unimanual clinical assessments (MACS, Jebsen–Taylor Hand Function Test (JTHFT), [23, 26, 29, 42, 48]) and 5 used bimanual assessments (AHA, Abilhand-Kids, Children’s Hand-use Experience Questionnaire (CHEQ) [24, 25, 28, 29, 35, 42]).

Among those that used unimanual clinical assessments, no correlation was found between MACS level and movement time or kinematic values [42]. Moderate correlations were found between JTHFT and goal synchronisation (r = 0.634, p < 0.05), and total task duration (r = 0.39, p < 0.05) [29].

Among those that used bimanual clinical assessments, the highest correlation was found between the AHA score and Arm Profile Score (APS) (a kinematic index) (r = − 0.84, P < 0.001) [24]. Low to moderate correlations were found between AHA score and bilateral movement measures (total movement time and goal synchronisation) (r = -0.3 p < 0.05) [28] and no correlation was found for normalized movement overlap [28, 35]. The AHA “smoothness of movement” item was moderately and significantly correlated with the SPARC and IOC quality of movement parameters [23]. Poor to good significant correlations were found between Abilhand-Kids and the maximum angular value (MAX) and range of motion (ROM) (r = 0.36–0.58, p < 0.03) [25, 42], and movement time (r = 0.769, p = 0.001) [42]. CHEQ sub-scores for the affected hand were significantly correlated with total task duration (r = 0.41–51, p < 0.05), temporal coupling (r = 0.36, p < 0.05) and spatial accuracy (r = 0.41–0.59; p < 0.05) [29].

#### Discriminant validity

The mean COSMIN score of the studies that assessed discriminant validity was 8/9 points.

Twelve studies compared children and adolescents with uCP and TDC [23–25, 31, 33, 34, 36, 37, 39, 42, 43, 48]. The main results were that children with uCP had restricted ROM of shoulder elevation, plane of elevation, elbow extension, supination, wrist extension and wrist adduction/abduction, and vertical and lateral hand movements compared to TDC during bimanual tasks [24, 25, 36, 37, 39, 42, 48].

Children and adolescents with uCP had altered spatiotemporal variable values, with longer movement durations [31, 37, 42, 48] and lower peak velocities [39, 48] compared to TDC. Children and adolescents with uCP used their hands less often at the same time (less goal-synchronized), with less interlimb coupling [33, 34] than TDC. Hand trajectories were less smooth [24, 31, 43, 48] and also less straight [24] in children and adolescents with uCP. The duration of use of each UL was more asymmetrical in children with uCP because the affected UL was underused as compared with TDC [23].

#### Reliability

The mean COSMIN score of the studies that assessed reliability was 14/15 points.

Reliability was evaluated in 2 studies, either within-session [24] or both within and between sessions [25]. These studies explored 2 different versions of a protocol (‘Be An Airplane Pilot’ (BE API) and BE API 2.0). Within session reliability was assessed during 3 or 4 movement cycles for each task. Between-session reliability was assessed at an interval of 2 and 4 weeks. Reliability was assessed with correlated multiple correlations (CMC), intraclass correlation coefficients (ICC) and measurement errors (minimum detectable change [MDC] and standard error of measurement [SEM]). Within- and between-session reliability were high for kinematic variables: CMC > 0.82; ICC > 0.85, SEM 4.78° and moderate for smoothness and trajectory (ICC > 0.53).

#### Responsiveness

The mean COSMIN score of the studies that assessed responsiveness was 8/12 points.

The main objective of the studies was to evaluate the effect of an intervention but not specifically the responsiveness of the measures. Seven studies performed assessments pre- and post-intervention: Constraint-Induced Movement Therapy (CIMT) [30, 35], Hand and Arm Bimanual Intensive Therapy (HABIT) [26, 35, 38], structured practice [27] and synchronized metronome training [40, 41]. All outcome measures changed significantly post-intervention except after CIMT [30]. After HABIT, goal synchronization time (p < 0.05) [26, 35], movement overlap (p = 0.005) [35] and % time of bimanual movements (p = 0.001) increased [38], and kinematic variables improved (trunk displacement decreased [26%, p < 0.05] and UL joint excursion [30%, p < 0.01] and elbow extension increased [25%, p < 0.05] on the affected side) [38]. Similar findings were reported for structured/unstructured practice although kinematic variables only improved for structured practice [27]. Two studies reported significant changes in smoothness, distance and ROM of elbow, wrist and shoulder after metronome training [40, 41].

### Synthesis of outcome measures evaluated for clinical and research purposes

Spatiotemporal variables were evaluated both for research and clinical practice. Bilateral variables were used for research [28, 38, 44, 47, 49–52] and clinical practice (pre-post intervention [27, 35, 37]) but only one study evaluated convergent validity: goal synchronization was correlated with the unimanual JTHFT assessment [33]).

The outcome measures that underwent the most validity assessments were ROM, MAX, APS, SPARC and IOC (convergent, discriminative validity and reliability) for research purposes [24, 25]. Accelerometery variables (mean activity count and asymmetry index) were used for convergent, discriminative validity and pre-post therapy, both in research and clinical settings [23, 26]. Kinematic (ROM), spatiotemporal variables (peak velocity, movement time, number of movement units and movement duration) [27, 30, 35, 37, 40, 41, 48] and quality of movement variables (smoothness) were used to assess the effect of therapy.

## Discussion

This systematic review of studies that used instrumented assessments of bimanual movements, most often 3DMA, in children and young adults with uCP aged from 2 to 25 years, found that the majority of studies were of good to very good quality (mean Q score 22/32). The protocols evaluated were varied, involving 1 to 5 bimanual tasks that mostly represented activities of daily living or were part of a game scenario. Spatiotemporal variables were most often evaluated, including 6 variables specifically developed for the assessment of bimanual movement: task completion time, goal synchronization, movement overlap time, interlimb coupling, continuous relative phase and asynchrony. The instrumented measures demonstrated moderate to good discriminant and convergent validity, but reliability and responsiveness assessments were lacking. All types of variables discriminated between children and adolescents with CP and TDC and were used to assess UL therapy efficacy with relevant results, therefore demonstrating their potential for clinical and research purposes.

### Types of instruments

A wide variety of instruments has been used to quantitatively evaluate bimanual movements, depending on the objective or variables of interest (ex: accelerometers for actimetry). Most studies used 3DMA, probably because this was the first tool to be developed and because it has been shown to be consistent and accurate for gait analysis, for which it has become the gold standard [11]. However, the 3DMA system set-up for UL recordings appears to lack consensus with regard to the number of cameras, markers, etc. 3DMA is performed in a laboratory setting to measure standardized, reliable movements that may differ from those performed in daily life. We recommend the use of 3DMA as it provides an objective and accurate measure, which complements the clinical assessment but with more technical standardization. The results of this review also highlighted that other recently developed technologies, which involve smaller and less constraining systems, e.g. accelerometers, can be used [23, 26]. However, no studies have yet used these instruments to provide out-of-laboratory assessments and to investigate bimanual movements directly in daily situations (e.g. at home), likely because real-life measures are less standardized, require greater tolerance for longer recordings, and the analysis of bimanual data from the real-life setting is challenging [13]. Accelerometers or inertial measurement units should be increasingly used to measure UL performance in the home setting, i.e., how the child spontaneously uses their impaired UL in real life. Another pertinent suggestion is to adapt daily life objects to perform direct recordings in the child’s usual environment (e.g., toys, etc.) and to get away from the context of evaluation [41]. We recommend the development of tools that allow direct measurement of UL movements in real-life situations with as few constraints on the child as possible. This is necessary to improve understanding of the effects of therapies and to adapt them to the real difficulties encountered in daily life (Additional file 5).

### Protocols

The protocols lacked standardization, they involved different data collection procedures and, most importantly, different bimanual tasks. The choice of tasks for bimanual movement assessment is a real challenge because of the large variety of movements that can be performed with both ULs (symmetrical/asymmetrical, free/constrained, with/without object, proximal/distal, etc.). Hung et al. proposed a unique task, ‘the drawer-opening task’ [26–28, 33–35, 38], mainly to study coordination, however, most protocols used several tasks to provide a comprehensive and global assessment of bimanual movement. Based on the results of this review, we recommend the use of a set of 3 to 5 tasks both to provide an overview of the performance of different bimanual movements and to ensure precision, while also maintaining the attention and participation of the child during the assessment. To evaluate spontaneous movements as they are performed in daily life, tasks that involve interaction with objects should be evaluated: bimanual UL movement is often induced by the need to grasp, manipulate or hold objects. Some studies particularly focused on interaction with objects (size and shape) [34, 39, 51, 53] and factors that interfered with interaction and how information was processed (visual disturbance) [31, 44–46] to evaluate all the situations that the child may face in daily life and to contribute to the understanding of bimanual function. In laboratory conditions, we recommend the assessment of simple daily movements (e.g., dressing, drinking and holding) or tasks within a game scenario [24, 25, 32, 48, 53] to better reflect spontaneous bimanual movements, whilst still being reproducible. The environment could be enhanced by video games or virtual reality [47]. The results of the review also support the need to directly assess bimanual movement in real-life situations (at home or school) (Additional file 5).

### Outcome measures

Spatiotemporal variables were the most often analysed. They were mainly used to describe the characteristics of bimanual movements and to evaluate the effect of therapies. Six bilateral variables were specifically developed to assess bilateral movements. Most bilateral variables were spatiotemporal and were designed for the ‘drawer-opening task’ [26–28, 33–35, 38] whereas other bilateral variables explored coordination between both hands [31, 44, 47, 53]. These variables may constitute important indicators since children and adolescents with CP use different motor coordination patterns during daily bimanual activities. Moreover, understanding coordination patterns could guide researchers and clinicians in the development of intervention programs that aim to improve bimanual hand coordination performance [6]. However, these variables have only been partially validated; reliability and responsiveness have yet to be determined. With regards to UL kinematics, similar variables to those used in unimanual assessments have been used to assess each UL separately, often to compare the ULs, however, no variables have been specifically developed to evaluate joint angles during bimanual movements. Quality of movement variables, i.e., smoothness or straightness, provide information that is difficult to obtain precisely with clinical assessments; this is a real advantage of motion analysis. However, there is also no consensus on the best way to measure some of these quality of movement variables (e.g. smoothness was measured using 6 different methods [24, 30, 31, 40, 41, 43, 45, 47, 48, 53]) and none have been completely validated. This finding highlights the difficulty of determining relevant UL variables that could be assessed across all types of bimanual tasks. According to the results of this review, we recommend that a comprehensive assessment of UL impairment should include spatiotemporal, kinematic and quality of movement parameters, including specific bilateral variables (goal synchronization, movement overlap time, etc.). This would help to better characterize pathological movements and to further determine those that are most relevant to guide the focus of therapies in clinical practice (Additional file 5).

### Validation of instrumented measures

The results of this review showed that most measures have not been fully validated. Reliability and responsiveness properties have not been sufficiently assessed, despite their importance. A representative example is the “drawer task”: it was used in 7 studies but only convergent and discriminant validity have been confirmed. Discriminant validity was the most evaluated: all the tools were able to differentiate between children with CP and TDC for all types of variables and bimanual tasks. Moreover, other types of discriminant validity were also evaluated, i.e. comparison between uni and bimanual movements [40, 41, 46, 47, 49–52] and comparison of the affected and non-affected ULs during asymmetrical tasks [29, 33, 40, 41]. Future studies should include an evaluation of reliability since we found only 2 studies that assessed this property [24, 25]. Given the complexity of UL movements and the use of different instruments/markers, reliability must be evaluated because factors such as the child’s position could impact the accuracy and interpretation of the measure. In this review, we were unable to properly report on the responsiveness of the tools as it was not the main objective of the studies included. However, most studies demonstrated changes in the values of variables after different interventions, whatever the protocol and variables assessed. Therefore, instrumented assessments appear to be good indicators of therapy efficacy. Regarding convergent validity, the highest correlations were found for assessments of the impaired UL during bimanual tasks, the AHA and APS (a kinematic deviation index of the impaired UL), whereas bilateral variables were not correlated, demonstrating the pertinence of evaluating both ULs to better reflect bimanual function.

### Limitations

This review has some limitations. The lack of standardization of protocols and systems made direct comparisons of results between studies challenging. The quality of the articles included ranged widely (from 13 to 30/32): full descriptions of data acquisition and adherence to biomechanical recommendations (e.g., International Society of Biomechanics) were frequently missing. Furthermore, no studies reported a priori sample size calculations, therefore the statistical power is unknown. Among the articles included, 8 were from the same team [26–28, 33–36, 38]. The quality scale developed was provided to help interpretation but has not been previously validated, therefore, the quality results should be interpreted with caution. The age range of the children and adolescents who were compared in this review was very wide: analysis by age is necessary as toddlers and young adults may have different bimanual function. The results could not be generalized to all children and adolescents with CP as there was a lack of assessment in younger children and those with bilateral CP. Future research, involving younger children (pre-schoolers), and taking into account the different degrees of UL impairment (MACS 3–5) and diversity of CP type (bilateral CP, dyskinetic), will improve our understanding of bimanual movements and their development in these children and adolescents (Additional file 5).

## Conclusion

This systematic review reported a large number of instrumented measurements of bimanual movements in children with CP, in good- to high-quality studies. These assessments involved different systems (3DMA, accelerometers, etc.) and different bimanual tasks (drawer-opening task, reach to grasp, etc.), and spatiotemporal parameters were mostly calculated in bimanual conditions. Some specific variables were developed for the evaluation of bimanual function (ex: goal synchronization). However, the metrological properties of these instruments were not fully evaluated, especially reliability and responsiveness. The complementary information provided by instrumented measures in relation to clinical assessments of bimanual function was highlighted. Development of other relevant variables and validation of these tools are required before they can be used as research outcomes or in clinical practice. Studies that involve younger children and real-life assessments will improve our understanding of bimanual function in these children.

## Supplementary Information

**Additional file 1: Table S1.** Search strategy used for systematic review.

**Additional file 2: Table S2.** ‘Design, Participants, Construct, Type of instruments, Outcome measures’ following the COSMIN standard for systematic reviews of Patient‐Reported Outcome Measures [21].

**Additional file 3.** Article quality was evaluated with a customized scale.

**Additional file 4: Table S3.** Results of the Q-score (quality assessment) of each article.

**Additional file 5.** Synthesis of recommendations for quantitative bimanual assessments.

## Data Availability

All data generated or analysed during this study are included in this published article and its Additional files.

## Abbreviations

3DMA: 3D motion analysis
AHA: Assisting hand assessment
APS: Arm profile score
BE API: Be an airplane pilot
CHEQ: Children’s Hand-use Experience Questionnaire
CIMT: Constraint-induced movement therapy
CMC: Correlated multiple correlations
COSMIN: Consensus based Standards for the selection of health Measurement Instruments
CP: Cerebral palsy
HABIT: Hand and arm bimanual intensive therapy
ICC: Intraclass correlation coefficients
IOC: Index of curvature
JTHFT: Jebsen–Taylor hand function test
MACS: Manual ability classification system
MAX: Maximum angular value
MDC: Minimum detectable change
ROM: Range of motion
SEM: Standard error of measurement
SPARC: Spectral arc length
TD: Typically developing
uCP: Unilateral cerebral palsy
UL: Upper limb
