# Supplementary material for: Bimanual movements in children with cerebral palsy: a systematic review of instrumented assessments
Source: J Neuroeng Rehabil. 2023 Feb 27;20:26. doi: 10.1186/s12984-023-01150-7 (PMC9972766; doi:10.1186/s12984-023-01150-7)
Supplement: Supplementary file 1 — Additional file 1: Table S1. Search strategy used for systematic review. [file 12984_2023_1150_MOESM1_ESM.pdf]

Additional File 1: Search strategy

		**Pubmed**		**Scopus**		**Web of science**		**Cochrane Library**	
**#**	**Text type**	**Search word**	**Hits**	**Search word**	**Hits**	**Search word**	**Hits**	**Search word**	**Hits**
1	Mesh	"Child"[Mesh] OR "Adolescent"[Mesh] OR "Infant"[Mesh]	3,691,623	/				Mesh Descriptor: [Child] OR Mesh Descriptor: [Adolescent] OR Mesh Descriptor [Infant]	125,202
2	Free text	Child*[text] OR Teen*[text] OR Infant [text] OR Adolescent*[text]	4,319,563	TITLE-ABS (child*) OR TITLE-ABS (teen*) OR TITLE-ABS (infant*) OR TITLE-ABS (adolescent*) OR TITLE-ABS (bab*) OR TITLE-ABS (newborn*) AND (LIMIT-TO (DOCTYPE , "ar"))	2,391,293	((((((((AB=(Child*)) OR AB=(teen*)) OR AB=(adolescent*)) OR AB=(infant*)) OR AB=(newborn*)) OR TI=(child*)) OR TI=(adolescent*)) OR TI=(teen*)) OR TI=(infant*)) OR TI=(newborn*)	2,229,231	(Child*):ti,ab, kw OR (Teen*):ti,ab, kw OR (Infant):ti,ab, kw OR (Adolescent*):ti,ab, kw OR (bab*):ti,ab, kw OR (newborn):ti,ab, kw	279,986
**3**		**#1 OR #2**	**4,319,563**		**2,391,293**	**#1 OR #2**	**2,229,231**	**#1 OR #2**	**279,986**
4	Mesh	"Upper Extremity "[Mesh]	171,813	/		/		Mesh Descriptor : [Upper Extremity]	7520
5	Free text	Upper Limb*[text] OR “Upper Extremit*”[text] Upper Bod*”[text] OR arm[text] OR bimanual[text] OR interlimb[text] OR hand*[text]	936,642	TITLE-ABS (upper AND limb*) OR TITLE-ABS (upper AND extremit*) OR TITLE-ABS (upper AND bod*) OR TITLE-ABS (arm*) OR TITLE-ABS (bimanual) OR TITLE-ABS (interlimb) OR TITLE-ABS (hand*) AND (LIMIT-TO (DOCTYPE , "ar"))	1,904,958	((((((((((((TI=(upper limb*)) OR TI=(upper extremit*)) OR TI=(upper body)) OR TI=(arm*)) OR TI=(bimanual)) OR TI=(interlimb*)) OR TI=(hand*)) OR AB=(upper limb*)) OR AB=(upper extremit*)) OR AB=(upper body)) OR AB=(bimanual)) OR AB=(interlimb*)) OR AB=(hand*)) OR AB=(arm*)	1,901,922	Upper Limb*[text] OR “Upper Extremit*”[text] Upper Bod*”[text] OR arm[text] OR bimanual[text] OR interlimb[text] OR hand*[text]	178,106
**6**		**#4 OR #5**	**1,004,247**		**1,904,958**	**#4 OR #5**	**1,901,922**	**#4 OR #5**	**180,608**
**7**		**#3 AND #6**	**168,436**	**#3 AND #6**	**102,894**	**#3 AND #6**	**77,724**	**#3 AND #6**	**27,731**
8	Mesh	"Movement"[Mesh:NoExp] OR "Motor Activity"[Mesh] OR"Movement disorders"[Mesh:noexp] OR"Motion"[Mesh]	776,381	/		/		"Movement"[Mesh:NoExp] OR "Motor Activity"[Mesh] OR"Movement disorders"[Mesh:noexp] OR"Motion"[Mesh]	12,415
9	Free text	"Movement"[text] OR Motor [text] OR Motion [text] OR "Kinematic*"[text] OR "Biomechanic*"[text] OR "Instrumented measurement"[text] OR "Inertial"[text] OR "Sensors"[text] OR "Acceleromet*"[text] OR " technolog*"[text] OR " spatio-temporal"[text] OR " temporo-spatial"[text] OR "smoothness"[text] OR “fluidity"[text] OR " trajectory"[text]	1,683,059	TITLE-ABS (movement) OR TITLE-ABS (motor) OR TITLE-ABS (motion) OR TITLE-ABS (kinematic*) OR TITLE-ABS (biomechanic*) OR TITLE-ABS (instrumented AND measurement) OR TITLE-ABS (inertial) OR TITLE-ABS (sensors) OR TITLE-ABS (acceleromet*) OR TITLE-ABS (technolog*) OR TITLE-ABS (spatio-temporal) OR TITLE-ABS (temporo-spatial) OR TITLE-ABS (smoothness) OR TITLE-ABS (fluidity) OR TITLE-ABS (trajectory) AND (	4,360,550	((((((((((((((((((((((((((((TI=(Movement)) OR TI=(Motor)) OR TI=(Motion)) OR TI=(Kinematic*)) OR TI=(Biomechanic*)) OR TI=(Instrumented measurement)) OR TI=(Inertial)) OR TI=(Sensor*)) OR TI=(Acceleromet*)) OR TI=(technolog*)) OR TI=(spatio-temporal)) OR TI=(temporo-spatial)) OR TI=(smoothness)) OR TI=(fluidity)) OR TI=(trajectory)) OR AB=(Movement)) OR AB=(Motor)) OR AB=(Motion)) OR AB=(Kinematic*)) OR AB=(Biomechanic*)) OR AB=(Instrumented	5,204,053	"Movement"[text] OR Motor [text] OR Motion [text] OR "Kinematic*"[text] OR "Biomechanic*"[text] OR "Instrumented measurement"[text] OR "Inertial"[text] OR "Sensor*"[text] OR "Acceleromet*"[text] OR " technolog*"[text] OR " spatio-temporal"[text] OR " temporo-spatial "[text] OR "smoothness"[text] OR “fluidity"[text] OR " trajectory"[text]	151,807

				LIMIT-TO (DOCTYPE , "ar"))		measurement)) OR AB=(Inertial)) OR AB=(Sensor*)) OR AB=(Acceleromet*)) OR AB=(technolog*)) OR AB=(spatio-temporal)) OR AB=(temporo-spatial)) OR AB=(smoothness)) OR AB=(fluidity)) OR AB=(trajectory)			
**10**		**#8 OR #9**	**2,023,656**		**4,360,550**	**#8 OR #9**	**5,204,053**	**#8 OR #9**	**154,958**
**11**		**#7 AND #10**	**22,881**	**#7 AND #10**	**13,790**	**#7 AND #10**	**12,506**	**#7 AND #10**	**5,140**
	Mesh	"Cerebral Palsy"[Mesh] OR "Hemiplegia"[Mesh] OR "Quadriplegia"[Mesh]	39,892	/		/		"Cerebral Palsy"[Mesh] OR "Hemiplegia"[Mesh] OR "Quadriplegia"[Mesh]	2,307
	Free text	Cerebral Palsy [text] OR Hemipleg*[text] OR Hemiparesis [text] OR Quadripleg*[text] OR Tetrapleg*[text] OR Stroke[text] OR "Cerebrovascular accident" [text]	376,232	TITLE-ABS (cerebral AND palsy) OR TITLE-ABS (hemipleg*) OR TITLE-ABS (hemiparesis) OR TITLE-ABS (quadripleg*) OR TITLE-ABS (tetrapleg*) OR TITLE-ABS (stroke) OR TITLE-ABS (cerebrovascular AND accident) AND (LIMIT-TO (DOCTYPE , "ar"))	313,799	(((((((((((((TI=(hemipleg*)) OR TI=(cerebral palsy)) OR TI=(hemiparesis)) OR TI=(quadripleg*)) OR TI=(tetrapleg*)) OR TI=(stroke)) OR TI=(cerebrovascular accident)) OR AB=(cerebral palsy)) OR AB=(hemiparesis)) OR AB=(hemipleg*)) OR AB=(quadripleg*)) OR AB=(tetrapleg*)) OR AB=(stroke)) OR AB=(cerebrovascular accident)	361,541	Cerebral Palsy [text] OR Hemipleg*[text] OR Hemipares* [text] OR Quadripleg*[text] OR Tetrapleg*[text] OR Stroke[text] OR "Cerebrovascular accident" [text]	66,637
**14**		**#12 OR #13**	**376,250**	**#12 OR #13**	**313,799**	**#12 OR #13**	**361,541**	**#12 OR #13**	**66,637**
**15**		**#11 AND #14**	**2,209**	**#11 AND #14**	**1,923**	**#11 AND #14**	**1,753**	**#11 AND #14**	**959**
**Combined**			**6,844**						
