# Supplementary material for: Bimanual movements in children with cerebral palsy: a systematic review of instrumented assessments
Source: J Neuroeng Rehabil. 2023 Feb 27;20:26. doi: 10.1186/s12984-023-01150-7 (PMC9972766; doi:10.1186/s12984-023-01150-7)
Supplement: Supplementary file 2 — Additional file 2: Table S2. ‘Design, Participants, Construct, Type of instruments, Outcome measures’ following the COSMIN standard for systematic reviews of Patient‐Reported Outcome Measures [21]. [file 12984_2023_1150_MOESM2_ESM.pdf]

Additional File 2: Inclusion criteria

DESIGN	PARTICIPANTS	CONSTRUCT	TYPE OF INSTRUMENTS	OUTCOME MEASURES
• Full papers: Randomized controlled trials, case-control studies, single case, case series, etc.<br>• Written in English<br>• No systematic reviews nor conference papers	• Children, toddlers, adolescents<br>• Uni or bilateral cerebral palsy<br>• 0-18 years	Instrumental measurements of bimanual movements	• 3D motion analysis<br>• Accelerometers, sensors, inertial measurement units, etc.<br>• Other instruments	**Quantitative measures (motion analysis)**<br><br>• Kinematics (angular values)<br>• Movement quality (smoothness, straightness, etc.)<br>• Spatio-temporal / Temporo-spatial (velocity, acceleration, etc.)<br>• Accelerometry

'Design, Participants, Construct, Type of instruments, Outcome measures' following the COSMIN standard for systematic reviews of Patient-Reported Outcome Measures (Mokkink et al., 2018).
