# Supplementary material for: Bimanual movements in children with cerebral palsy: a systematic review of instrumented assessments
Source: J Neuroeng Rehabil. 2023 Feb 27;20:26. doi: 10.1186/s12984-023-01150-7 (PMC9972766; doi:10.1186/s12984-023-01150-7)
Supplement: Supplementary file 3 — Additional file 3. Article quality was evaluated with a customized scale. [file 12984_2023_1150_MOESM3_ESM.pdf]

Additional File 3: Quality score

1 Aims and hypothesis clearly stated: clear (2), partial (1), no (0)
2 Was participant consensus obtained before the study?: yes (2), no/not stated (0)
3 Description of the participant recruitment: clear (2), partial (1), no (0)
4 Description of the sample: clear (2), partial (0.5-1.5), no (0)
5 Data acquisition: clear (2), partial (1), no (0)
6 Description of the movement analysis system (3DMA, sensors, etc.): clear (2), partial (1), no (0)
7 Description of marker locations: clear (2), partial (1), no (0)
8 Description of the movement tasks: clear (2), partial (1), no (0)
9 Data analysis (biomechanical model, method, software): clear (2), partial (1), no (0)
10 Main outcomes of the study clearly stated? clear (2), partial (1), no (0)
11 Description of the gold standard: clear (2), partial (1), no (0)
12 Statistical analysis: clear (2), partial (1), no (0)
13 Sample size calculation: yes (2), no (0)
14 Results interpretable (statistical significance)? yes (2), partial (1), no (0)
15 Description of study limits: yes (2), partial (1), no (0)
16 Key findings answer the initial objectives (conclusion clearly stated): yes (2), partial (1), no (0)
