# Supplementary material for: Bimanual movements in children with cerebral palsy: a systematic review of instrumented assessments
Source: J Neuroeng Rehabil. 2023 Feb 27;20:26. doi: 10.1186/s12984-023-01150-7 (PMC9972766; doi:10.1186/s12984-023-01150-7)
Supplement: Supplementary file 4 — Additional file 4: Table S3. Results of the Q-score (quality assessment) of each article. [file 12984_2023_1150_MOESM4_ESM.pdf]

Additional File 4: Quality assessment of studies

	1	2	3	4	5	6	7	8	9	10	11	12	13	14	15	16	Total score	% score
Beani et al. 2020 [23]	2	2	2	2	2	2	2	1	1	2	1	1	0	2	2	2	**26**	**81**
Cacioppo et al. 2020 [24]	2	2	2	2	2	2	2	2	2	2	2	2	0	2	2	2	**30**	**94**
Cope et al. 2010 [30]	2	2	2	2	1	2	1	1	1	2	2	2	0	1	0	2	**23**	**75**
Feltham et al. 2010a [31]	1	2	1	2	2	2	2	2	1	1	0	2	0	2	0	2	**22**	**69**
Gaillard et al. 2019 [25]	2	2	2	2	2	2	2	2	2	2	2	2	0	2	2	2	**30**	**94**
Gordon et al. 2007 [26]	1	2	2	2	2	2	2	2	1	2	2	1	0	2	2	1	**26**	**81**
Howcroft et al. 2012b [32]	2	2	2	1	1	2	2	1	2	2	0	2	0	2	2	2	**25**	**78**
Hung et al. 2004 [33]	2	2	1	2	2	1	2	2	2	2	0	1	0	1	0	1	**21**	**66**
Hung et al. 2010 [34]	2	2	0	1	2	1	2	2	2	2	0	1	0	2	1	2	**22**	**69**
Hung et al. 2011 [35]	1	2	2	2	2	1	2	2	2	2	1	2	0	2	1	2	**25**	**78**
Hung et Meredith, 2014 [36]	1	2	1	2	2	2	2	2	2	2	0	1	0	2	0	2	**21**	**66**
Hung et al. 2017a. [27]	2	2	2	2	2	1	2	2	2	2	0	1	0	2	2	2	**26**	**81**
Hung et Spingarn, 2018 [37]	1	2	2	2	2	1	2	2	2	2	0	2	0	2	1	2	**25**	**78**
Hung et al. 2018 [38]	1	2	2	2	2	2	2	1	2	2	0	1	0	2	1	1	**23**	**75**
Hung et al. 2019 [28]	2	2	2	2	1	2	2	2	2	2	2	2	0	2	2	2	**29**	**91**
Hung et Zeng, 2020. [39]	2	2	2	2	1	2	2	2	2	2	0	1	0	2	1	2	**25**	**78**
Johansson et al. 2012 [40]	2	2	0	2	2	2	1	2	2	2	0	1	0	1	0	1	**20**	**63**
Johansson et al. 2014 [41]	2	2	1	2	2	2	1	2	2	2	0	1	0	1	2	1	**23**	**72**
Klotz et al, 2014 [42]	1	2	1	2	1	2	1	2	1	1	2	2	0	2	2	1	**23**	**72**
Mutalib et al, 2019a. [43]	1	2	0	2	1	2	2	2	2	2	0	2	0	2	0	1	**21**	**66**
Rudisch, et al, 2016 [29]	2	2	2	1	1	2	2	2	2	2	2	2	0	2	1	2	**26**	**81**
Shum et al, 2020 [48]	2	2	0	2	2	1	0	1	0	1	0	2	0	0	1	1	**15**	**47**
Smorenburg et al. 2011 [44]	2	2	1	2	1	2	2	2	0	2	0	1	0	1	1	1	**20**	**63**
Smorenburg et al, 2012a [45]	1	2	1	2	2	2	0	2	2	2	0	1	0	2	1	2	**22**	**69**
Smorenburg et al, 2013 [46]	1	2	1	2	2	2	0	2	1	1	2	1	0	2	0	2	**21**	**66**
Sugden et Utley, 1995 [49]	1	2	1	2	2	1	2	2	1	1	0	0	0	0	0	0	**15**	**47**
Utley et Sugden,1998. [50]	1	1	1	2	2	2	1	1	1	1	0	1	0	1	0	0	**15**	**47**
Utley et al. 2004 [51]	1	1	0	2	1	1	1	2	1	1	0	0	0	1	0	1	**13**	**41**
Utley et al. 2007 [52]	1	1	0	2	1	2	1	2	1	1	0	0	0	1	0	1	**14**	**44**
Van Thiel et al. 2001 [53]	1	0	1	1	1	2	1	2	2	1	0	1	0	1	0	1	**15**	**47**
Volman et al., 2020 [47]	2	2	2	2	2	2	2	2	2	2	0	1	0	2	0	2	**25**	**78**
