# Supplementary material for: Bimanual movements in children with cerebral palsy: a systematic review of instrumented assessments
Source: J Neuroeng Rehabil. 2023 Feb 27;20:26. doi: 10.1186/s12984-023-01150-7 (PMC9972766; doi:10.1186/s12984-023-01150-7)
Supplement: Supplementary file 5 — Additional file 5. Synthesis of recommendations for quantitative bimanual assessments. [file 12984_2023_1150_MOESM5_ESM.pdf]

**Additional File 5: Synthesis of recommendations for quantitative bimanual assessments**

## Recommendations for quantitative bimanual assessments

### Measurements systems

- 3D motion analysis
- Out-of-laboratory measures
    - Accelerometers, sensors
    - Connected devices

### Protocols

- 3-5 bimanual tasks
- Interaction with object (grasping, manipulating, holding)
- Different condition (speed, visual disturbance, etc.)
- Simple daily movements or playful situation
- Importance of environment
- Daily settings (home or school)

### Parameters

- Combination of:
- Kinematics
- Quality of movement

With specific bilateral measure for bimanual situation
